# Quantification of Volatile Metabolites Derived From Garlic (*Allium sativum*) in Human Urine

**DOI:** 10.3389/fnut.2019.00043

**Published:** 2019-04-16

**Authors:** Laura Scheffler, Constanze Sharapa, Andrea Buettner

**Affiliations:** ^1^Chair of Aroma and Smell Research, Department of Chemistry and Pharmacy, Emil Fischer Center, Friedrich-Alexander-Universität Erlangen-Nürnberg, Erlangen, Germany; ^2^Department Sensorical Analytics, Fraunhofer Institute for Process Engineering and Packaging (IVV), Freising, Germany

**Keywords:** gas chromatography-mass spectrometry, stable isotope dilution assay (SIDA), allyl methyl sulfide, allyl methyl sulfoxide, allyl methyl sulfone, human urine

## Abstract

The consumption of garlic (*Allium sativum*) is widely known to (negatively) impact body odor, in particular breath and sweat, but also urine. Despite this common phenomenon, the underlying processes in the body that lead to the malodor are not yet fully understood. In previous studies we identified three volatile garlic-derived metabolites in human milk and urine, namely allyl methyl sulfide (AMS), allyl methyl sulfoxide (AMSO), and allyl methyl sulfone (AMSO_2_). In the present study, we monitored the excretion processes of these metabolites via human urine after consumption of garlic over time, whereby 19 sets of eight urine samples (one sample pre-ingestion and seven samples post-ingestion) were analyzed using two-dimensional high resolution gas chromatography-mass spectrometry/olfactometry (HRGC-GC-MS/O). The highest concentrations of these metabolites were detected in urine ~1–2 h after garlic ingestion, with a second increase observed after 6–8 h in the urine of some participants. Moreover, the highest observed concentrations differed between the individual participants or test series by up to one order of magnitude.

## Introduction

Garlic (*Allium sativum*) is used since centuries as a condiment to refine the taste of dishes. However, garlic intake is often followed by a change in body odor, being commonly recognized in breath (1), sweat (2) but also in human milk (3–5). Besides its typical aroma, garlic is also known for its beneficial health properties, including antimicrobial and immunostimulating effects, protective effects against cancer and reduction of the risk of cardiovascular diseases (6). The health promoting effects as well as the impact on the body odor are mostly associated with the organosulfur compounds being major constituents of garlic, like the thiosulfinate allicin and its various degradation products (e.g., diallyl disulfide, diallyl trisulfide, ajoene) (7–10). However, food components, including volatiles, can be strongly modified by metabolism and biotransformation processes within the human body (11–13). Recent research has shown, that such transformations of aroma substances are not only limited to the liver but can already take place within the gastrointestinal tract (14) or during absorption processes (15). This can either lead to a loss of the respective bioactivity but can also result in the formation of newly generated bioactive compounds. In our previous studies, we identified three garlic-derived metabolites, namely allyl methyl sulfide (AMS), allyl methyl sulfoxide (AMSO), and allyl methyl sulfone (AMSO_2_) in human milk as well as in urine (5, 16). Based on these observations, the aim of the present study was to quantify these metabolites in human urine, specifically to characterize differences in the metabolite excretion between different test persons, and to monitor their temporal development over the course of excretion within a time period of up to 24 h. To allow for better comparison between individuals, test persons consumed portions of one garlic bulb in each case, and urine profiles were tested in parallel.

## Materials and Methods

### Chemicals

Ammonium chloride (NH_4_Cl), dichloromethane (DCM), sodium chloride (NaCl), and anhydrous sodium sulfate (Na_2_SO_4_) were purchased from VWR (Darmstadt, Germany). DCM was freshly distilled prior to use. The reference substances AMS and AMSO_2_, creatinine, disodium hydrogen phosphate (Na_2_HPO_4_), sodium sulfite (NaSO_3_) and urea were obtained from Aldrich (Steinheim, Germany). The reference standard AMSO as well as the isotopically labeled standards ^2^H_3_- AMS, ^2^H_3_-AMSO, and ^2^H_3_-AMSO_2_ were supplied by Aromalab (Freising, Germany). Garlic (white garlic, origin: Spain) was purchased from local supermarkets (Aldi-Süd, Erlangen, Germany or Rewe, Freising, Germany), whereas the creatinine kit was from Labor+Technik Eberhard Lehmann GmbH (Berlin, Germany).

### Human Urine Samples

Human urine samples were obtained from 18 volunteers (age 22–38 years, mean 27 years; 11 females, 7 males); thereby, one volunteer conducted the whole experimental series twice. At the time of examination, volunteers reported no known illness that might have potentially influenced the metabolism or the excretion of urine. The test persons were instructed to avoid foods that were rich in sulfur-containing substances at the testing day as well as 2 days preceding the testing day, namely garlic, onion, wild garlic, chives, cabbage, and leek, and were only allowed to consume the garlic sample provided in the course of the experiments. Additionally, subjects were asked to keep records of the food (including food supplements) and beverages they consumed during these 3 testing days. Generally, the overall panelists' requirements were in accordance with our previous studies (5, 16).

On the sampling day, the outer garlic cloves of a whole garlic bulb were peeled and cut into ~3 mm cubes by using a garlic cutter (Genius GmbH, Limburg/Lahn, Germany). Subsequently these cubes were thoroughly mixed and aliquoted into 3 g portions. Three to four test persons conducted the experiment at the same day. Each test person was asked to ingest a garlic portion of 3 g. In total, eight consecutive urine samples were provided by each test person, comprising one whole urine set. The samples were collected in sterile brown glass bottles, with sampling times as follows: one sample was provided directly prior to garlic consumption. The series of the second to eighth sample was then obtained at about 0.5, 1, 2, 4, 6, 8, and 24 h after garlic consumption, respectively. Between the first urine sample (prior to garlic consumption) and the seventh urine sample (8 h after garlic consumption) test persons only spent urine at the specific time points mentioned before. Another sample was provided at the next morning (24 h after garlic consumption). Between the samples provided 8 and 24 h after garlic consumption no urine was collected. All samples were immediately placed into a −80°C freezer and were kept frozen until further analysis.

The samples of each set were termed according to their sampling time. The following list provides the time intervals that are related to the respective sample name:

- *pre*: before garlic consumption
- *0.5 h post*: 0.4–0.8 h after garlic consumption
- *1 h post*: 0.9–1.3 h after garlic consumption
- *2 h post*: 1.8 h−2.2 h after garlic consumption
- *4 h post*: 3.8–4.4 h after garlic consumption
- *6 h post*: 5.8–6.4 h after garlic consumption
- *8 h post*: 7.8–8.4 h after garlic consumption
- *24 h post*: 23.8–25.6 h after garlic consumption

The first sample of each set was tested with a dipstick-test, the multiple test stripes “Combi-Screen PLUS” (Analyticon Biotechnologies AG, Lichtenfels, Germany), to check the health status of each test person, allowing for simultaneous testing of ascorbic acid, bilirubin, blood, glucose, ketones, leucocytes, nitrite, pH, protein, specific gravity/density, and urobilinogen in the respective urine samples.

### Determination of the Creatinine Concentration in Urine Samples

A creatinine kit (Labor+Technik Eberhard Lehmann GmbH, Berlin, Germany) was used to determine the creatinine content in each urine sample. The determination principle of this kit is based on the reaction of creatinine with picric acid under basic conditions (Jaffé reaction). The formed complex was detected photometrically at a wavelength of 492 nm.

### Solvent Assisted Flavor Evaporation (SAFE) of Volatiles From Human Urine

For quantification purposes, the deuterated standards ^2^H_3_-AMS, ^2^H_3_-AMSO, and ^2^H_3_-AMSO_2_ were added to the sample and stirred for 10 min. Subsequently, DCM was added in a ratio of 1:2 (DCM/human urine; v/v) and this solution was stirred for further 30 min at room temperature. To isolate the volatile fraction from the urine samples we then applied SAFE-distillation (17), thereby distilling the respective samples at 50°C. After completion of the distillation process, 10 mL of DCM were applied in each case to rinse the SAFE apparatus and to ensure complete transition of the volatile compounds. This process was repeated with another 10 mL of DCM. After thawing, the obtained aqueous distillate phase was extracted three times with 25 mL DCM. The organic phases were combined, dried over anhydrous Na_2_SO_4_ and concentrated to a total volume of 100 μL by means of Vigreux distillation and subsequent micro-distillation at 50°C.

### High Resolution Gas Chromatography-Mass Spectrometry (HRGC-MS)

GC-MS analysis was performed with an Agilent MSD quadrupole system (GC 7890A and MSD 5975C, Agilent Technologies, Waldbronn, Germany) equipped with a GERSTEL MPS 2 auto sampler and a GERSTEL CIS 4 injection system (GERSTEL, Duisburg, Germany). A DB-FFAP (30 m × 0.25 mm, film thickness 0.25 μm, Agilent J&W Scientific, Santa Clara, CA, USA) was used as analytical capillary. An uncoated, deactivated fused silica capillary (2–3 m × 0.53 mm) was used as pre-column. This capillary was changed regularly to avoid accumulation of impurities. A further uncoated fused silica capillary (0.3–0.6 m × 0.25 mm) was used as a transfer line into the MS. Carrier gas was Helium at a flow rate of 1.0 mL/min. Mass spectra were recorded at 70 eV in Selected Ion Monitoring (SIM)-mode (cf. Table 1) for quantification. To verify the identity of the compounds, their mass spectra were additionally recorded in full scan mode (mass-to-charge ratio (*m/z*) range 30–350). The following temperature program for the oven was used: 40°C was held for 7 min. This temperature was raised to 240°C at a rate of 8°C/min. The final temperature was held for 8 min. Injection volume was 2 μL. The sample was applied with the auto sampler using the cold-on-column technique (18)

**Table 1:** Parameters of the quantification method including the instrument which was used for measurement, selected ions of analytes, and isotopically labeled standards and calibration factors.

**Instrument**	**Analyte**		**Standard**		**Calibration function**	***R*^**2**^**
	**Compound**	**m/z**	**Compound**	**m/z**		
GC-GC-MS	AMS	88	^2^H_3_- AMS	91	y = 0.9386x−0.0153	0.9994
GC	AMSO	104	^2^H_3_- AMSO	107	y = 0.9883x+0.0062	0.9999
GC	AMSO_2_	120	^2^H_3_- AMSO_2_	123	y = 0.9935x+0.0264	0.9999

### Two-Dimensional High Resolution Gas Chromatography-Mass Spectrometry/Olfactometry (HRGC-GC-MS/O) (Heart-Cut)

For quantification of AMS a two-dimensional GC-MS system was applied. The system consisted of two Agilent 7890 B GCs coupled with an Agilent 5977 B MS (Agilent, Waldbronn, Germany). The system was equipped with a GERSTEL MPS 2 auto sampler and a GERSTEL CIS 4 injection system (GERSTEL, Duisburg, Germany). A multi-column switching system μMCS was installed in the first GC and both GCs were connected via a cryogenic-trap system CTS 1 (both: GERSTEL, Duisburg, Germany). DB-5 (30 m × 0.32 mm, film thickness 0.25 mm; Agilent J&W Scientific, Santa Clara, CA, USA; first oven) and DB-FFAP (30 m × 0.32 mm, film thickness 0.25 mm; Agilent J&W Scientific, Santa Clara, CA, USA; second oven) were used as analytical capillaries. An uncoated, deactivated fused silica capillary was used as pre-column (2–3 m × 0.53 mm) as described previously. Helium was used as carrier gas at a constant flow rate of 2.5 mL/min in the first GC and 1.0 mL/min in the second GC. With the help of the μMCS, the effluent was split in the first oven between a flame ionization detector (FID) and an olfactory detection port (ODP 3, GERSTEL), as well as a cryotrap during cut intervals. In the second oven the effluent was split between an ODP and the MS by using a Y-splitter. All split capillaries were made of uncoated, deactivated fused silica material. The FID and both ODPs were held at 250° and 270°C, respectively. Mass spectra were recorded at 70 eV in full scan mode (*m/z* range 30–100) as well as in SIM-mode (cf. Table 1). 2 μL of the sample were injected on-column using the auto sampler. The temperature programs were as follows: starting temperatures were 40°C for both GCs. In the first GC this temperature was held for 8 min, in the second oven for 7 min. Thereafter it was raised to 300°C in the first oven and to 240°C in the second oven at a rate of 20°C/min. The final temperatures were held for 5 min. The transfer line between first and second oven was kept at 250°C, and during cut intervals it was cooled down to −100°C with liquid nitrogen.

### Quantification by Stable Isotope Dilution Assay (SIDA)

In pretest studies the amounts of AMS, AMSO and AMSO_2_ in urine samples after garlic ingestion were evaluated (data not shown). The respective isotopically labeled standards were dissolved in DCM and added to the urine samples collected before and after garlic ingestion according to the expected amounts based on these precedent trials. The mixture was worked up as described above. Quantification of AMSO and AMSO_2_ was performed by GC-MS analyses in SIM mode. The *m/z*-ratios 104 + 107 and 120 + 123 were selected for analysis of AMSO and AMSO_2_ and their respective labeled analogs. The quantification of AMS was carried out by GC-GC-MS analysis in SIM mode. The selected *m/z*-ratios for AMS and its labeled standard were 88 and 91. For quantification of the garlic-derived metabolites calibration curves were prepared consisting of defined mixtures of analyte standards and their respective isotopic labeled standards (w/w; AMS/ ^2^H_3_-AMS: 1:10, 1:5, 1:3, 1:2, 1:1, 2:1; AMSO/^2^H_3_-AMSO and AMSO_2_/^2^H_3_-AMSO_2_: 1:10, 1:5, 1:2, 1:1, 2:1, 3:1, 5:1). Calibration curves were calculated as functions of the intensity ratios of standard to labeled standard and their respective mass ratios (cf. Table 1). Calibration curves were prepared in triplicates, and at three different days. For quantification the average of these calibration curves was used. With the resulting calibration function, the known amount of isotopic labeled standard that was added to the sample, and the intensity ratio of analyte to isotopic labeled standard, the amount of the analyte in the sample was calculated.

### Calculation of Metabolite Profiles

The concentrations of the metabolites were calculated as μg/kg urine by dividing the calculated amount of the metabolites [in μg, cf. section Quantification by Stable Isotope Dilution Assay (SIDA)] by the amount of the respective urine sample (in kg). Additionally, the concentrations were normalized to the creatinine content of the respective urine sample by dividing the amount of the analyte by the volume of the respective urine sample (in L), which was further divided by the creatinine concentration of the respective sample (in mmol/L). As a result, the dilution effects attributed to different water intake of the test persons does not influence the absolute obtained excretion profile of the metabolites so that the excretion profiles of different test persons can be directly compared.

### Determination of the Limits of Detection (LOD) and Quantification (LOQ)

LOD and LOQ were determined according to the calibration line method described in DIN 32645. To ensure consistent conditions for the analyses of LOD and LOQ, synthetic urine instead of human urine was used. The formula of the synthetic urine was described by Mayrovitz and Sims (19). Unlabeled and labeled reference compounds were added to the synthetic urine in the concentration range of LOD and LOQ. Subsequently the samples were worked up as described above (cf. section Solvent Assisted Flavor Evaporation (SAFE) of Volatiles From Human Urine) and analyzed by means of GC-MS and GC-GC-MS as described in sections High Resolution Gas Chromatography-Mass Spectrometry (HRGC-MS) and Two-Dimensional High Resolution Gas Chromatography-Mass Spectrometry/Olfactometry (HRGC-GC-MS/O) (Heart-Cut). As the provided urine samples were highly variable with regard to their volume (19–50 mL), LOD, and LOQ were calculated as absolute amounts (in ng).

## Results

The volatile garlic-derived metabolites were quantified in human urine after test persons consumed a defined amount of raw garlic (3 g) in order to characterize the metabolism and excretion processes of volatile garlic components in humans. The target metabolites, namely AMS, AMSO, and AMSO_2_, had been identified in our previous study when comparing urine samples collected before garlic consumption with urine samples collected after garlic consumption (16). In the current study, these garlic-derived metabolites were quantified in urine samples from different subjects collected over a time period of about 24 h. In the majority of cases these metabolites were only detected in samples that were gathered after garlic consumption. Only in a few cases (U*rine III, VII, VIII, XI, XII, XVI*, and *XVII*) they were also present in samples collected prior to garlic intervention, however only small amounts were detected in these cases (AMS ≤ 0.2 μg/mmol creatinine, AMSO ≤ 1.4 μg/mmol creatinine, AMSO_2_ ≤ 2.6 μg/mmol creatinine). Presumably, these test persons consumed foods during the wash out phase that contained sulfur-bearing constituents such as garlic or onion. Based on these observations, the food protocols of all participating test persons were checked. According to the food protocols of the test persons providing U*rine III, VII, VIII, XI, XII, XVI*, and *XVII*, they reported that they had consumed foods from restaurants, like roasted pork leg, lamb meat, doner kebab, filled pepper, stir-fried vegetable, or an arabic soup, but no food supplements. These foods can be assumed to have contained onion or garlic ingredients for seasoning, which might explain the presence of the metabolites in the respective control urine samples. However, as the test persons stayed at the institute at the sampling day, the consumed food of the test persons was controlled in this period of time, ensuring no additional entry of garlic sources, besides the garlic administered within the frame of the intervention. Despite the presence of AMS, AMSO, and AMSO_2_ in the control urine samples, we observed distinct increases in the subsequent samples collected after garlic intake.

**Figure 1:**
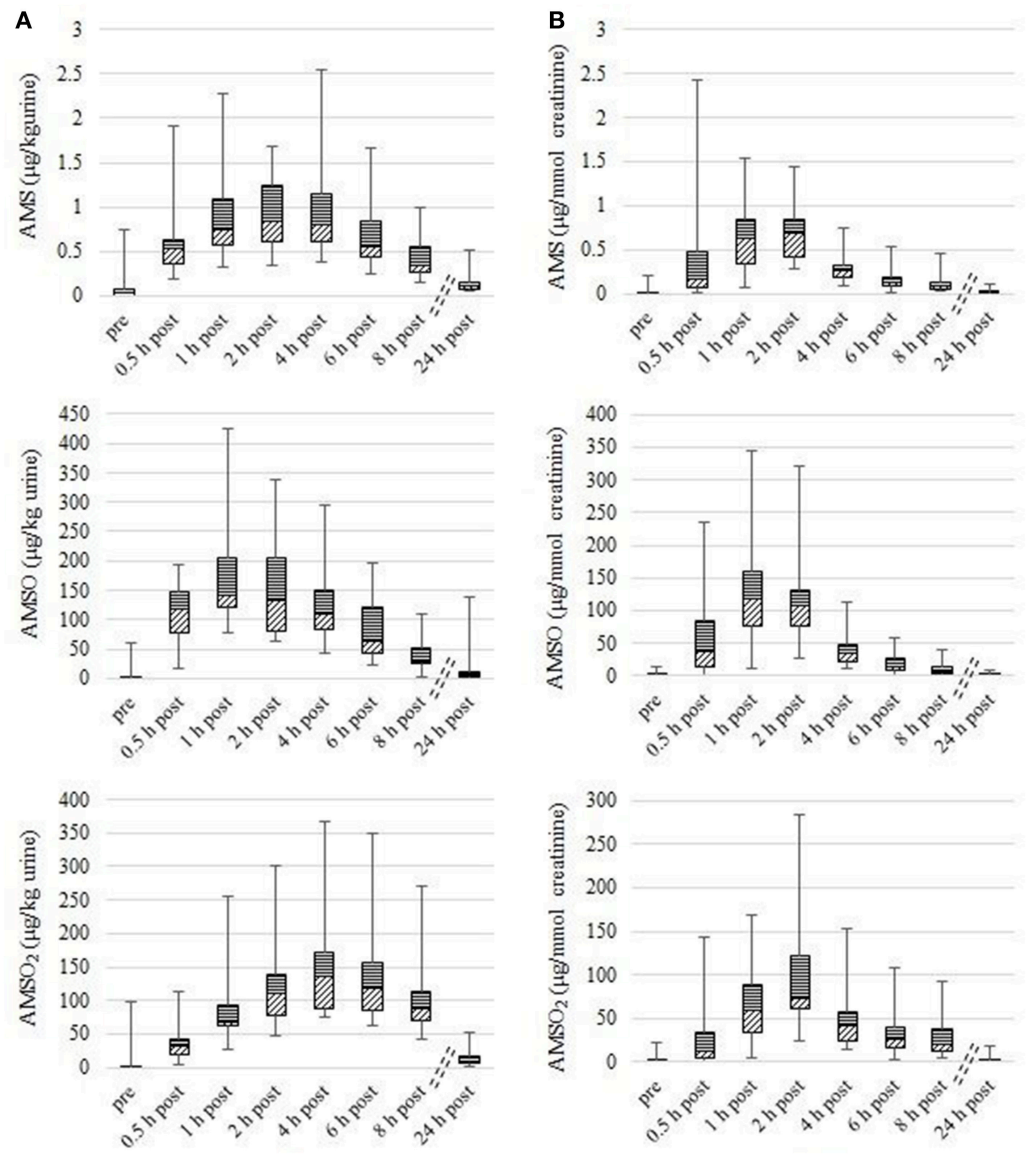
Box plot (mean value, markers at minimum and maximum metabolite concentration, box perc. 25–75%) of garlic-metabolite (AMS, AMSO, and AMSO_2_) concentrations in human urine, expressed as μg/kg **(A)** and μg/mmol creatinine **(B)**. Data compiles 18 urine sets. The term “pre” relates to the urine samples that were collected before garlic consumption. “0.5 h post” – “24 h post” relate to the urine samples that were collected after garlic intake.

The concentrations of the metabolites were calculated in μg/kg urine and in μg/mmol creatinine. The highest concentrations of AMS, AMSO and AMSO_2_ in a series of urine samples after garlic consumption varied in a range from 0.5 to 2.5 μg/kg urine, from 77.0 to 423.9 μg/kg urine, and from 75.1 to 367.0 μg/kg urine, respectively. As the metabolite concentration in urine depends on the water intake of the test person, the concentrations of AMS, AMSO, and AMSO_2_ were normalized to the creatinine content of the respective urine samples. Thereby, the normalized concentrations of the metabolite maxima ranged between 0.3 and 2.4 μg/mmol creatinine (AMS), 27.6 and 344.1 μg/mmol creatinine (AMSO), and between 32.1 and 284.7 μg/mmol creatinine (AMSO_2_), roughly comprising one order of magnitude in each case. Apart from these variations in the concentration range, the temporal appearance of the highest metabolite concentration differed to some extent between the individual urine sets. In most cases (17 out of 19 urine sets) the highest normalized AMS concentration was detected 1 or 2 h after garlic consumption. Only in one case (*Urine VIII*) the highest AMS amount was already detected 0.5 h after garlic ingestion, whereas in *Urine XVI* the highest AMS concentration was only detected 4 h after garlic consumption. The highest determined amounts of AMSO and AMSO_2_ were observed between 1 and 2 h after garlic ingestion in the urine samples with the sole exception of *Urine XVI* where the maximum of AMSO_2_ was observed as late as 4 h after garlic intake. In relation to the maximum of AMSO, the maximum of AMSO_2_ either appeared correspondingly at the same time point (as was the case for 14 urine sets), or with a temporal delay, namely in the subsequent sample (as was the case for 5 urine sets). Even 8 h after garlic consumption all metabolites were still present in quantifiable amounts in all samples, which means that all metabolites were above their respective LOQ (LOQ (AMS): 7.6 ng, LOQ (AMSO): 77.4 ng, LOQ (AMSO_2_): 78.6 ng). Only about 24 h after garlic consumption the metabolite concentrations in urine were reduced to traces in most of the urine sets. In the case of these samples the amounts ranged roughly between the LOQ and the LOD [LOD (AMS): 1.6 ng, LOD (AMSO): 22.0 ng, LOD (AMSO_2_): 22.4 ng]. The results are summarized in Figure 1 (metabolite concentration in μg/kg urine: Figure 1A; metabolite concentration in μg/mmol creatinine: Figure 1B).

**Figure 2:**
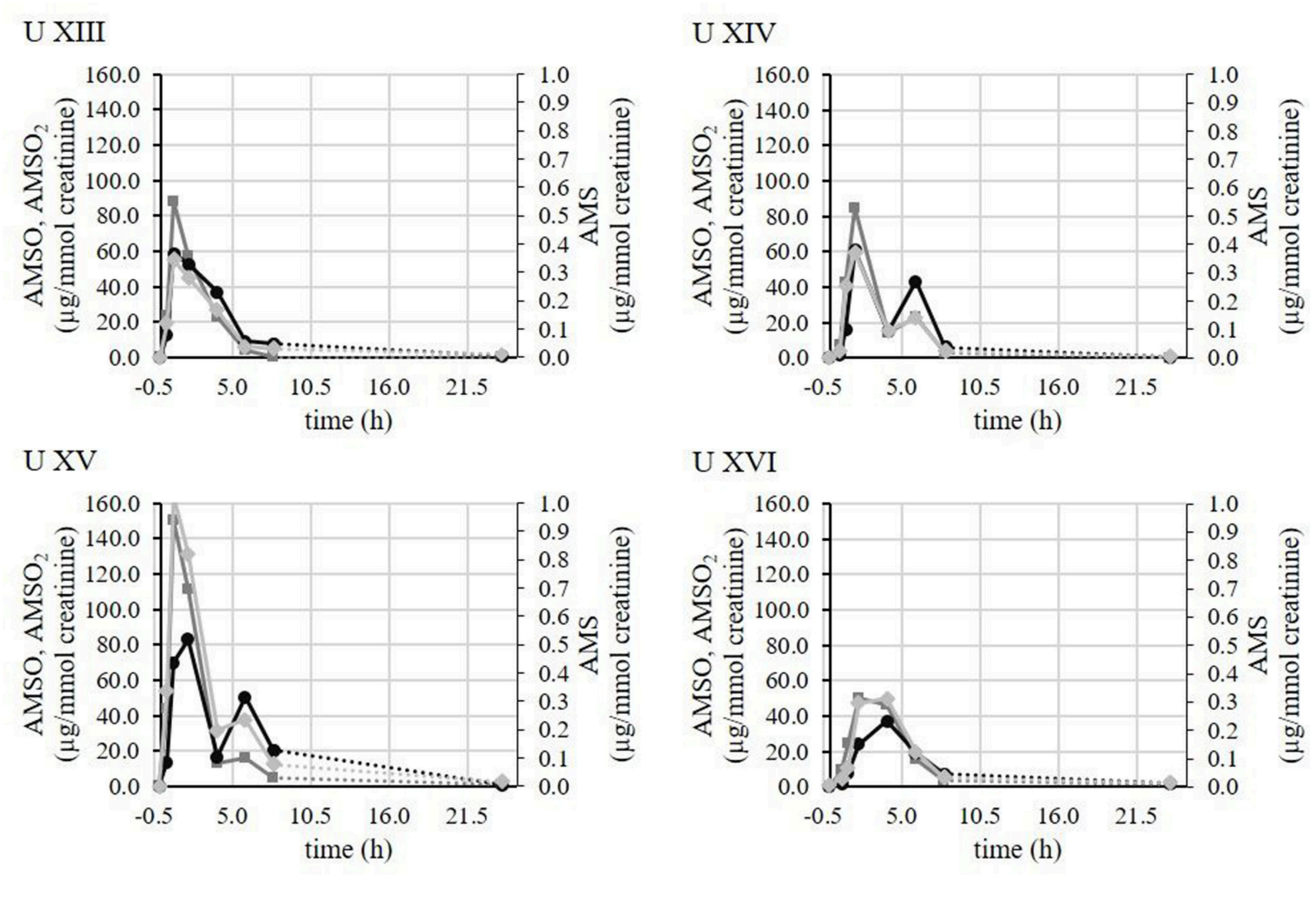
Time-resolved metabolite profiles of AMS, AMSO, and AMSO_2_ of four test persons (*Urine XIII, Urine XIV, Urine XV, Urine XVI*). All four test persons consumed a garlic sample from the same garlic bulb. Time 0 h corresponds to the time of garlic intake. (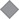) AMS; (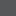) AMSO; (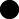) AMSO_2_. Broken line: time interval, when no sample was collected.

For better visualization representative time-resolved metabolite profiles of AMS, AMSO and AMSO_2_ are displayed in Figure 2 for four different test persons who conducted the experiment at the same day (*Urine XIII, Urine XIV, Urine XV*, and *Urine XVI*). Despite the fact that all test persons consumed 3 g of garlic that was taken from the same chopped and mixed garlic bulb, thereby ensuring that the composition of the garlic sample was comparable, we observed distinct differences in the metabolite profiles between samples. In these cases, the highest detected AMS concentrations of each sample series were 0.3 μg/mmol creatinine (*Urine XIII*), 0.4 μg/mmol creatinine (*Urine UIV*), 1.0 μg/mmol creatinine (*Urine XV*), and 0.3 μg/mmol creatinine (*Urine XVI*). Accordingly, the concentrations of the AMS maxima showed a variation by a factor of up to 3.2 between the individual test persons. Regarding AMSO the highest concentrations were 87.7 μg/mmol creatinine (*Urine XIII*), 84.7 μg/mmol creatinine (*Urine XIV*), 150.4 μg/mmol creatinine (*Urine XV*), and 50.1 μg/mmol creatinine (*Urine XVI*), yielding a variation factor of up to 3.0 between urine sets. Finally, in case of AMSO_2_, the concentrations were 58.5 μg/mmol creatinine (*Urine XIII*), 60.1 μg/mmol creatinine (*Urine XIV*), 83.1 μg/mmol creatinine (*Urine U XV*), and 37.19 μg/mmol creatinine (*Urine XVI*) corresponding to a factor of 2.2 between the highest and lowest concentration. Furthermore, differences in the temporal profiles of metabolite excretion were observed in these cases. In the cases of *Urine XIII* and *Urine XV* the concentration of each metabolite increased rapidly after garlic consumption and after reaching the maximum about 1 h (*Urine XIII*) or 2–4 h (*Urine XV*) after ingestion of garlic, the excreted metabolite concentration decreased continuously. In contrast to this, the sample sets of *Urine XIV* and *Urine XV* exhibited two maxima for each metabolite. The first maximum was observed in each case between 1 and 2 h after garlic consumption, whereas the second maximum was observed about 6 h after garlic consumption. With regard to the other samples, that are not visualized here, similar differences were observed for all test persons, regardless whether they conducted the experiment on the same day with garlic samples obtained from the same garlic bulb or on different test days. Consequently, the observed differences in metabolite profiles cannot be attributed to variations in the garlic samples only, but are obviously additionally linked to the individual physiological processes of the subjects. A compilation of all investigated urine samples, including time of urine sampling, mass (g), volume (mL), and creatinine content (mmol/L) as well as the concentrations of AMS, AMSO, and AMSO_2_ in μg/mL and μg/mmol creatinine are provided in the Table S1.

## Discussion

In our previous study (20) we quantified volatile garlic-derived metabolites in human milk; there, mothers consumed 3 g of raw garlic, which is in correspondence with the present study. However, the concentrations determined in the current study for urine were about twice as high in case of AMSO_2_ and about three times as high in case of AMSO. Only AMS revealed comparable excretion levels in both media. These findings generally agree with the fact that water soluble compounds are primarily excreted via urine (21) as AMSO and AMSO_2_ are more polar than AMS. Accordingly, it is likely that urinary excretion is preferred to the excretion via human milk. Regarding the temporal progress of the excretion only a limited comparison is possible. Since milk samples need to be provided according to the established lactation routine of the nursing mothers in order to not disturb the lactation rhythm, the sampling times are often not comparable. Yet, the highest concentrations of garlic-derived metabolites were usually detected in the first milk sample after garlic consumption, which corresponds to a time window of about 1–3 h after garlic intake. Likewise, a maximum was observed in case of the urine samples about 1–2 h after garlic consumption, demonstrating corresponding kinetics.

As described in our previous studies on the identification of garlic-derived compounds in human milk and urine, the detected thio compounds are likely to originate from allicin (5, 16). Allicin itself is formed from alliin, an odorless S-allyl-L-cysteine sulfoxide, due to the action of the enzyme *alliinase* after disruption of the cell structure (22). Allicin can be further converted into various compounds, e.g., dithiins, sulfides, polysulfides, and ajoenes (23, 24). As shown by Lawson and Wang (25) they can react with cysteine that is present in the intestinal tract and form S-allylmercaptocysteine. However, there is also evidence that S-allylmercaptoglutathione is formed on the basis of glutathione, either due to an enzymatic conjugation with diallyl disulfide or due to a non-enzymatic reaction with allicin (26). In both cases allyl mercaptan is formed subsequently. Allyl mercaptan can be methylated which leads to the formation of AMS.

Studies performed by Lawson and Wang (27) confirmed that compounds comprising a dithioallyl-group (e.g., allicin, diallyl disulfide, and ajoene) as well as AMS itself are transformed within the human body to AMS with allyl mercaptan as a precursor. However, an accumulation of this precursor was not expected due to a rapid methylation. In the current study we could confirm the formation of AMS. Furthermore, we could demonstrate that AMS is accompanied by AMSO and AMSO_2._ These findings are also in agreement with a previous study where authors identified AMSO and AMSO_2_ in rat urine and plasma after administration of diallyl disulfide (28).

The fate of garlic compounds that do not comprise a dithioallyl group, which is the case for diallyl sulfide or dithiins, is not yet fully resolved. Lawson and Wang (27) suggested diallyl sulfone as a metabolite for diallyl sulfide. This compound has been identified in rats after administration of diallyl sulfide (29). However, in our previous studies we could exclude the presence of this metabolite in human urine and human milk (5, 16), and again confirmed this finding in the present study. It is possible that diallyl sulfide is transformed to less volatile metabolites, such as glutathione or acetyl-conjugates (30, 31) and hence not detectable with the methods used in this study. Likewise, this could be the metabolic fate of the dithiins.

The current study aimed at quantifying volatile garlic-derived metabolites in human urine over a time interval of up to about 24 h. As a result it could be concluded that the impact of garlic on human urine composition differed between test persons. The variations included differences in the concentrations as well as differences in the temporal excretion pattern of the excreted metabolites. These findings are shown exemplarily in the time-resolved metabolite profiles of four test persons (*Urine XIII*- *XVI*) (cf. Figure 2). Although all test persons consumed garlic samples of the same garlic bulb, distinct differences in their metabolite profiles were observed. In this way it could be ruled out, that the variations between the test persons originated from different garlic composition. Possible reasons for the observed differences in excreted metabolite concentrations could comprise, amongst others, differences in the absorption processes that can be influenced by e.g., the individual resorptive area and the blood circulation and transport mechanisms therein. Amongst others, previous studies of our group could show that chemosensorially active compounds can be strongly biotransformed in the gastrointestinal tract, and even at the resorptive sites (14, 15, 32). Enzymes that are involved in the metabolism of garlic constituents could further exhibit genetic polymorphisms or differential expression rates which could lead to different plasma concentrations and therefore also urine concentrations; this has been reported for several enzymes responsible in drug metabolism (33). Moreover, we observed different temporal excretion pattern. In particular we observed maxima for AMS, AMSO, and AMSO_2_ that commonly occurred about 1–2 h after garlic ingestion. However, in some cases there was a second maximum additionally about 6–8 h after garlic consumption. This indicates that absorption and metabolism processes may take place at different sites in the human body. The rapid detection of garlic-derived metabolites in the urine samples already 0.5–1 h after garlic ingestion suggests an early uptake of garlic constituents. Although foods are primarily crushed in the oral cavity, which is the first step in digestion, the oral cavity might already be the first stage along the gastro-intestinal tract to absorb some of the food components. Such effects have been observed in case of drugs (34, 35). In case of volatiles, which are addressed in the present study, an absorption via the nasal epithelial cells could also lead to an early metabolite appearance in the urine samples (36, 37). Absorption processes have further been reported in the stomach for different drugs and plant pigments (38, 39). Accordingly, they are not restricted to the small intestine, but can take place throughout the whole gastro-intestinal tract. Apart from that, the storage time of urine in the bladder may further influence the excretion profiles of the compounds. In case of the observed second maximum, for example, it might be the case that due to mixing effects the second increase might become less pronounced in some urine samples compared to others or may even coincide with the first maximum. Regarding the physiological effects of garlic, it is interesting to note, that neither AMSO nor AMSO_2_ are odor-active components (5), and only AMS comprises a garlic-like aroma (5). Therefore, AMS might contribute to the changed body odor which is often recognized after garlic consumption. Furthermore, Khatua et al. (40) recently showed a correlation between AMS and AMSO and cardiac hypertrophy in rats. By increasing the Na^+^/K^+^-ATPase these metabolites attenuated cardiac hypertrophy. Therefore, they could influence the progression of cardiovascular diseases (41). However, potential effects in humans still remain to be proven.

## Conclusion

The impact of garlic consumption on human urine composition was investigated. Garlic-derived metabolites, namely AMS, AMSO, and AMSO_2_ were quantified via SIDA over a period of 24 h. Large inter-individual differences were observed, both with regard to the metabolite concentrations and ratios in the urine samples, as well as their excretion kinetics. The highest observed concentrations of AMS, AMSO and AMSO_2_ at the time point when the excretion maximum was reached ranged from 0.3 to 2.4 μg/mmol creatinine, 28 to 344 μg/mmol creatinine, and 32 to 285 μg/mmol creatinine, respectively. Commonly, the highest concentrations were found to occur about 1–2 h after garlic consumption, however for some urine sets a second increase was observed about 6–8 h after garlic consumption. These findings suggest differences in the absorption, distribution, metabolism, and excretion processes of volatile garlic constituents in the human body.

## Ethics Statement

The study was conducted in agreement with the Declaration of Helsinki. Written, informed consent was given by all 18 volunteers prior to the testing day. Withdrawal from the study was possible at any time. The study (registration number 49_13B) was approved by the Ethical Committee of the Medical Faculty, Friedrich-Alexander Universität Erlangen-Nürnberg.

## Author Contributions

LS, CS, and AB conceived and designed the experiments. LS performed the experiments and analyzed the data. AB contributed reagents, materials, and analysis tools. LS and CS conceived the publication that was approved by AB. All authors have read and approved the final manuscript.

### Conflict of Interest Statement

The authors declare that the research was conducted in the absence of any commercial or financial relationships that could be construed as a potential conflict of interest.

## Abbreviations

AMS: Allyl methyl sulfide
AMSO: Allyl methyl sulfoxide
AMSO_2_: Allyl methyl sulfone
DCM: Dichloromethane
HRGC-GC-MS/O: Two-dimensional high-resolution gas chromatography-mass spectrometry/olfactometry
HRGC-MS: High-resolution gas chromatography-mass spectrometry
FID: Flame ionization detector
LOD: Limit of detection
LOQ: Limit of quantification
*m/z* ratio: Mass-to-charge ratio
ODP: Olfactory detection port
SAFE: Solvent-assisted flavor evaporation
SIDA: Stable isotope dilution assay
SIM: Selected ion monitoring.

## References

[1] Suarez F Springfield J Furne J Levitt M. Differentiation of mouth versus gut as site of origin of odoriferous breath gases after garlic ingestion. Am J Physiol Gastrointest Liver Physiol. (1999) 276:G425–G430. 10.1152/ajpgi.1999.276.2.G425 9950816

[2] Doty RL Olfactory communication in humans. Chem Senses. (1981) 6:351–76. 10.1093/chemse/6.4.351

[3] Mennella JA Beauchamp GK. Maternal diet alters the sensory qualities of human milk and the nurslings's behavior. Pediatrics. (1991) 88:737–44. 1896276

[4] Mennella JA Beauchamp GK. The effects of repeated exposure to garlic-flavored milk on the nursling's behavior. Pediatr Res. (1993) 34:805–8. 10.1203/00006450-199312000-00022 8108198

[5] Scheffler L Sauermann Y Zeh G Hauf K Heinlein A Sharapa C. Detection of volatile metabolites of garlic in human breast milk. Metabolites. (2016) 6:18. 10.3390/metabo6020018 27275838 PMC4931549

[6] Rose P Whiteman M Moore PK Zhu YZ. Bioactive S-alk(en)yl cysteine sulfoxide metabolites in the genus Allium: the chemistry of potential therapeutic agents. Nat Prod Rep. (2005) 22:351–68. 10.1039/b417639c 16010345

[7] Apitz-Castro R Ledezma E Escalante J Jain MK. The molecular basis of the antiplatelet action of ajoene: direct interaction with the fibrinogen receptor. Biochem Biophys Res Commun. (1986) 141:145–50. 10.1016/S0006-291X(86)80346-1 3800991

[8] Choi YH Park HS. Apoptosis induction of U937 human leukemia cells by diallyl trisulfide induces through generation of reactive oxygen species. J Biomed Sci. (2012) 19:50. 10.1186/1423-0127-19-50 22578287 PMC3404941

[9] Borlinghaus J Albrecht F Gruhlke MCH Nwachukwu ID Slusarenko AJ. Allicin: chemistry and biological properties. Molecules. (2014) 19:12591–618. 10.3390/molecules190812591 25153873 PMC6271412

[10] Bradley JM Organ CL Lefer DJ. Garlic-derived organic polysulfides and myocardial protection. J Nutr. (2016) 146:403S–9S. 10.3945/jn.114.208066 26764335 PMC4725427

[11] Horst K Rychlik M. Quantification of 1,8-cineole and of its metabolites in humans using stable isotope dilution assays. Mol Nutr Food Res. (2010) 54:1515–29. 10.1002/mnfr.200900528 20425757

[12] Kirsch F Horst K Röhrig W Rychlik M Buettner A Tracing metabolite profiles in human milk: studies on the odorant 1,8-cineole transferred into breast milk after oral intake. Metabolomics. (2012) 9:483–96. 10.1007/s11306-012-0466-9

[13] Schaffarczyk M Balaban TS Rychlik M Buettner A Syntheses of chiral 1,8-cineole metabolites and determination of their enantiomeric composition in human urine after ingestion of 1,8-Cineole-containing capsules. ChemPlusChem. (2013) 78:77–85. 10.1002/cplu.201200253

[14] Heinlein A Buettner A. Monitoring of biotransformation of hop aroma compounds in an *in vitro* digestion model. Food Funct. (2012) 3:1059–67. 10.1039/c2fo30061c 22740026

[15] Heinlein A Metzger M Walles H Buettner A. Transport of hop aroma compounds across Caco-2 monolayers. Food Funct. (2014) 5:2719–30. 10.1039/C3FO60675A 24977259

[16] Scheffler L Sauermann Y Heinlein A Sharapa C Buettner A. Detection of volatile metabolites derived from garlic (*Allium sativum*) in human urine. Metabolites. 6:E43. 10.3390/metabo6040043 27916960 PMC5192449

[17] Engel W Bahr W Schieberle P Solvent assisted flavour evaporation—a new and versatile technique for the careful and direct isolation of aroma compounds from complex food matrices. Eur Food Res Technol. (1999) 209:237–41. 10.1007/s002170050486

[18] Schomburg G Behlau H Dielmann R Weeke F Husmann H Sampling techniques in capillary gas chromatography. J Chromatogr A. (1977) 142:87–102. 10.1016/S0021-9673(01)92028-X

[19] Mayrovitz HN Sims N. Biophysical effects of water and synthetic urine on skin. Adv Skin Wound Care. (2001) 14:302–8. 10.1097/00129334-200111000-00013 11794441

[20] Scheffler L Sharapa C Buettner A. Quantification of volatile metabolites derived from garlic in human breast milk. Food Chem. (2019) 274:603–10. 10.1016/j.foodchem.2018.09.039 30372984

[21] Eaton DL Gallagher EP General overview of toxicology. In: McQueen CA, editor. Comprenesive Toxicology. Kidlington: Elsevier (2010). p. 1–46.

[22] Block E The organosulfur chemistry of the genus allium—implication for the organic-chemistry of sulfur. Angew Chem Int. (1992) 31:1135–78. 10.1002/anie.199211351

[23] Iberl B Winkler G Knobloch K. Products of allicin transformation—ajoenes and dithiins, characterization and their determination by hplc. Planta Med. (1990) 56:202–11. 17221396 10.1055/s-2006-960926

[24] Lawson LD Wang ZYJ Hughes BG. Identification and HPLC quantitation of the sulfides and dialk(en)yl thiosulfinates in commercial garlic products. Planta Med. (1991) 57:363–70. 10.1055/s-2006-960119 1775579

[25] Lawson LD Wang ZJ Pre-hepatic fate of the organosulfur compounds derived from garlic (*Allium sativuin*). Planta Med. (1993) 59(S 1):A 688 10.1055/s-2006-959976

[26] Germain E Chevalier J Siess MH Teyssier C. Hepatic metabolism of diallyl disulphide in rat and man. Xenobiotica. (2003) 33:1185–99. 10.1080/00498250310001636840 14742141

[27] Lawson LD Wang ZJ. Allicin and allicin-derived garlic compounds increase breath acetone through allyl methyl sulfide: use in measuring allicin bioavailability. J Agric Food Chem. (2005) 53:1974–83. 10.1021/jf048323s 15769123

[28] Germain E Auger J Ginies C Siess MH Teyssier C. *in vivo* metabolism of diallyl disulphide in the rat: identification of two new metabolites. Xenobiotica. (2002) 32:1127–38. 10.1080/0049825021000017902 12593760

[29] Brady JF Ishizaki H Fukuto JM Lin MC Fadel A Gapac JM. (1991). Inhibition of cytochrome P-450 2E1 by diallyl sulfide and its metabolites. Chem. Res. Toxicol. (2016) 4, 642–647. 10.1021/tx00024a008 1807447

[30] de Rooij BM Boogaard PJ Rijksen DA Commandeur JNM Vermeulen NPE. Urinary excretion of N-acetyl-S-allyl-L-cysteine upon garlic consumption by human volunteers. Arch Toxicol. (1996) 70:635–9. 10.1007/s002040050322 8870956

[31] Jin JX Baillie TA. Metabolism of the chemoprotective agent diallyl sulfide to glutathione conjugates in rats. Chem Res Toxicol. (1997) 10:318–27. 10.1021/tx9601768 9084912

[32] Müller J Gruner N Almstätter I Kirsch F Buettner A Pfaffl MW Investigation into the metabolism of 1,8-cineole in an intestinal cell culture model and acquisition of its immune-modulatory effect via gene expression analysis. Flavour Fragr J. (2012) 27:405–13. 10.1002/ffj.3109

[33] Evans WE Relling MV. Pharmacogenomics: Translating Functional Genomics into Rational Therapeutics. Science. (1999) 286:487–91. 10.1126/science.286.5439.487 10521338

[34] Harris D Robinson JR. Drug delivery via the mucous membranes of the oral cavity. J Pharm Sci. (1992) 81:1–10. 10.1002/jps.2600810102 1619560

[35] Rathbone MJ Drummond BK Tucker IG The oral cavity as a site for systemic drug delivery. Adv Drug Deliv Rev. (1994) 13:1–22. 10.1016/0169-409X(94)90024-8

[36] Keyhani K Scherer PW Mozell MM. A Numerical Model of Nasal Odorant Transport for the Analysis of Human Olfaction. J Theor Biol. (1997) 186:279–301. 10.1006/jtbi.1996.0347 9219668

[37] Linforth R Martin F Carey M Davidson J Taylor AJ. Retronasal Transport of Aroma Compounds. J Agric Food Chem. (2002) 50:1111–7. 10.1021/jf011022n 11853491

[38] Hogben CAM Schanker LS Tocco DJ Brodie BB Absorption of drugs from the stomach. II The human Journal of Pharmacology and Experimental Therapeutics. (1957) 120:540–5. 13476378

[39] Passamonti S Vrhovsek U Vanzo A Mattivi F. The stomach as a site for anthocyanins absorption from food. FEBS Lett. (2003) 544:210–3. 10.1016/S0014-5793(03)00504-0 12782318

[40] Khatua TN Borkar RM Mohammed SA Dinda AK Srinivas R Banerjee SK Novel Sulfur Metabolites of Garlic Attenuate Cardiac Hypertrophy and Remodeling through Induction of Na+/K+-ATPase Expression. Front Pharmacol. (2017) 8:18 10.3389/fphar.2017.00018 28194108 PMC5276815

[41] Kumar S Kumar B Kumar R Kumar S Khatkar SK Kanawjia SK Nutritional features of goat milk—a review. Ind J Dairy Sci. (2012) 65:266–73.

