# Supplementary material for: Quantification of Volatile Metabolites Derived From Garlic (Allium sativum) in Human Urine
Source: Front Nutr. 2019 Apr 16;6:43. doi: 10.3389/fnut.2019.00043 (PMC6499206; doi:10.3389/fnut.2019.00043)
Supplement: Supplementary file 1 [file Table_1.DOCX]

Supplementary Material

Quantification of Volatile Metabolites derived from Garlic
(Allium sativum) in Human Urine

Laura Scheffler^1^, Constanze Sharapa^1^, Andrea Buettner ^1,2,*^

*** Correspondence:** Professor Dr. Andrea Buettner: andrea.buettner@fau.de

# Supplementary Table

Table S1: Compilation of investigated urine samples: time of urine sampling, amount of consumed garlic (g), mass (g) and volume (mL) of the investigated sample as well as AMS, AMSO and AMSO_2_ (µg/kg and µg/mmol creatinine) of the urine samples.

| sample | |  | sampling time |  | consumed garlic |  | quantity  urine sample | |  | AMS | |  | AMSO | |  | AMSO_2_ | |
| --- | --- | --- | --- | --- | --- | --- | --- | --- | --- | --- | --- | --- | --- | --- | --- | --- | --- |
|  |  |  | (h) |  | (g) |  | (g) | (mL) |  | (µg/kg) | (µg/mmol creatinine) |  | (µg/kg) | (µg/mmol creatinine) |  | (µg/kg) | (µg/mmol creatinine) |
| U I^a^ | pre |  | -0.03 |  | 3.1217 |  | 25.0726 | 25 |  | < LOD^1^ | < LOD^1^ |  | < LOD^2^ | < LOD^2^ |  | < LOD^3^ | < LOD^3^ |
|  | 0.5 h post |  | 0.55 |  |  |  | 50.5441 | 50 |  | 0.9 | 0.1 |  | 168.4 | 23.6 |  | 41.7 | 5.8 |
|  | 1 h post |  | 1.03 |  |  |  | 49.9672 | 50 |  | 0.7 | 0.8 |  | 191.0 | 205.9 |  | 98.5 | 106.3 |
|  | 2 h post |  | 2.05 |  |  |  | 50.1499 | 50 |  | 1.7 | 0.8 |  | 227.6 | 112.5 |  | 138.8 | 68.4 |
|  | 4 h post |  | 4.05 |  |  |  | 37.5975 | 37 |  | 1.6 | 0.2 |  | 165.7 | 19.1 |  | 172.3 | 19.6 |
|  | 6 h post |  | 6.05 |  |  |  | 50.8882 | 50 |  | 1.5 | 0.2 |  | 119.1 | 13.2 |  | 170.7 | 18.6 |
|  | 8 h post |  | 8.05 |  |  |  | 50.8192 | 50 |  | 0.7 | 0.1 |  | 41.8 | 5.8 |  | 119.9 | 16.3 |
|  | 24 h post |  | 25.55 |  |  |  | 50.8383 | 50 |  | traces^1^ | traces^1^ |  | 1.6 | 0.3 |  | 5.6 | 0.9 |

Tabelle S1: continued

| U II^a^ | pre |  | -0.13 | |  | 3.023 |  | 51.041 | 50 |  | < LOD^1^ | < LOD^1^ |  | < LOD^2^ | < LOD^2^ |  | < LOD^3^ | < LOD^3^ |
| --- | --- | --- | --- | --- | --- | --- | --- | --- | --- | --- | --- | --- | --- | --- | --- | --- | --- | --- |
|  | 0.5 h post |  | 0.50 | |  |  |  | 35.5639 | 35 |  | 0.5 | 0.1 |  | 147.7 | 14.0 |  | 32.4 | 3.0 |
|  | 1 h post |  | 1.00 | |  |  |  | 50.0888 | 50 |  | 0.9 | 0.3 |  | 133.4 | 51.2 |  | 68.0 | 26.1 |
|  | 2 h post |  | 2.00 | |  |  |  | 50.0055 | 50 |  | 0.5 | 0.4 |  | 63.7 | 62.6 |  | 62.1 | 61.0 |
|  | 4 h post |  | 4.00 | |  |  |  | 50.3614 | 50 |  | 0.7 | 0.1 |  | 64.8 | 12.0 |  | 75.1 | 13.8 |
|  | 6 h post |  | 6.00 | |  |  |  | 50.6888 | 50 |  | 0.4 | 0.04 |  | 33.4 | 3.7 |  | 61.7 | 6.8 |
|  | 8 h post |  | 8.00 | |  |  |  | 50.6741 | 50 |  | 0.2 | 0.02 |  | 15.2 | 1.8 |  | 41.3 | 4.9 |
|  | 24 h post |  | 24.12 | |  |  |  | 45.6525 | 45 |  | traces^1^ | traces^1^ |  | traces^2^ | traces^2^ |  | 1.9 | 0.1 |
| U III^a^ | pre |  | -0.03 | |  | 3.1324 |  | 40.6544 | 40 |  | traces^1^ | traces^1^ |  | 4.6 | 0.8 |  | 7.3 | 1.3 |
|  | 0.5 h post |  | 0.53 | |  |  |  | 50.2727 | 50 |  | 0.6 | 0.3 |  | 124.4 | 74.5 |  | 41.9 | 24.9 |
|  | 1 h post |  | 1.07 | |  |  |  | 50.0363 | 50 |  | 0.6 | 0.8 |  | 136.4 | 192.7 |  | 83.3 | 117.5 |
|  | 2 h post |  | 2.18 | |  |  |  | 50.2157 | 50 |  | 1.2 | 0.7 |  | 214.7 | 133.1 |  | 145.2 | 89.7 |
|  | 4 h post |  | 4.35 | |  |  |  | 50.3506 | 50 |  | 0.8 | 0.3 |  | 123.7 | 47.0 |  | 168.2 | 63.4 |
|  | 6 h post |  | 6.35 | |  |  |  | 50.6702 | 50 |  | 0.6 | 0.1 |  | 62.3 | 12.2 |  | 134.9 | 26.1 |
|  | 8 h post |  | 8.43 | |  |  |  | 50.6764 | 50 |  | 0.4 | 0.1 |  | 29.0 | 5.2 |  | 86.7 | 15.3 |
|  | 24 h post |  | 23.90 | |  |  |  | 45.7524 | 45 |  | traces^1^ | traces^1^ |  | 3.0 | 0.5 |  | 5.6 | 0.9 |
| U IV^b^ | pre |  | | -0.05 |  | 3.1578 |  | 32.5839 | 32 |  | < LOD^1^ | < LOD^1^ |  | < LOD^2^ | < LOD^2^ |  | < LOD^3^ | < LOD^3^ |
|  | 0.5 h post |  | | 0.55 |  |  |  | 50.2938 | 50 |  | 0.7 | 0.1 |  | 144.3 | 30.7 |  | 31.0 | 6.6 |
|  | 1 h post |  | | 1.05 |  |  |  | 50.0118 | 50 |  | 0.7 | 0.5 |  | 141.0 | 117.5 |  | 63.2 | 52.7 |
|  | 2 h post |  | | 2.12 |  |  |  | 50.0722 | 50 |  | 0.7 | 0.4 |  | 129.7 | 82.3 |  | 82.8 | 52.5 |
|  | 4 h post |  | | 4.05 |  |  |  | 50.4339 | 50 |  | 0.8 | 0.2 |  | 108.9 | 22.6 |  | 108.4 | 22.3 |
|  | 6 h post |  | | 6.08 |  |  |  | 51.0355 | 50 |  | 0.6 | 0.04 |  | 63.5 | 4.6 |  | 96.7 | 6.9 |
|  | 8 h post |  | | 8.05 |  |  |  | 50.299 | 50 |  | 0.3 | 0.1 |  | 31.9 | 6.7 |  | 73.4 | 15.4 |
|  | 24 h post |  | | 24.03 |  |  |  | 50.6691 | 50 |  | 0.5 | 0.1 |  | 3.8 | 0.4 |  | 6.8 | 0.7 |

Tabelle S1: continued

| U V^b^ | pre |  | -0.05 | |  | 3.1196 |  | 50.0426 | 50 |  | < LOD^1^ | < LOD^1^ |  | < LOD^2^ | < LOD^2^ |  | < LOD^3^ | < LOD^3^ |
| --- | --- | --- | --- | --- | --- | --- | --- | --- | --- | --- | --- | --- | --- | --- | --- | --- | --- | --- |
|  | 0.5 h post |  | 0.50 | |  |  |  | 49.9376 | 50 |  | 0.5 | 0.7 |  | 84.4 | 123.5 |  | 33.9 | 49.6 |
|  | 1 h post |  | 1.00 | |  |  |  | 49.9577 | 50 |  | 0.7 | 0.8 |  | 122.6 | 146.3 |  | 80.4 | 96.0 |
|  | 2 h post |  | 2.00 | |  |  |  | 49.9878 | 50 |  | 0.7 | 0.8 |  | 104.6 | 124.9 |  | 111.3 | 132.8 |
|  | 4 h post |  | 4.00 | |  |  |  | 50.1838 | 50 |  | 0.9 | 0.3 |  | 104.9 | 34.0 |  | 142.9 | 46.1 |
|  | 6 h post |  | 6.00 | |  |  |  | 50.308 | 50 |  | 0.7 | 0.1 |  | 68.8 | 13.8 |  | 151.3 | 30.1 |
|  | 8 h post |  | 8.00 | |  |  |  | 50.095 | 50 |  | 0.3 | 0.2 |  | 29.0 | 13.4 |  | 99.4 | 45.8 |
|  | 24 h post |  | 24.00 | |  |  |  | 50.1124 | 50 |  | traces^1^ | traces^1^ |  | 1.9 | 0.3 |  | 5.1 | 0.9 |
| U VI^b^ | pre |  | -0.05 | |  | 3.1545 |  | 50.5869 | 50 |  | < LOD^1^ | < LOD^1^ |  | < LOD^2^ | < LOD^2^ |  | < LOD^3^ | < LOD^3^ |
|  | 0.5 h post |  | 0.50 | |  |  |  | 50.0106 | 50 |  | 1.1 | 0.5 |  | 193.1 | 88.3 |  | 74.1 | 33.9 |
|  | 1 h post |  | 1.00 | |  |  |  | 44.9474 | 45 |  | 0.7 | 0.6 |  | 203.5 | 169.6 |  | 87.6 | 73.1 |
|  | 2 h post |  | 2.00 | |  |  |  | 49.9775 | 50 |  | 1.0 | 0.9 |  | 143.4 | 129.2 |  | 136.6 | 123.2 |
|  | 4 h post |  | 4.00 | |  |  |  | 50.1689 | 50 |  | 1.2 | 0.3 |  | 134.9 | 39.0 |  | 150.1 | 43.2 |
|  | 6 h post |  | 6.00 | |  |  |  | 50.2921 | 50 |  | 0.9 | 0.2 |  | 188.9 | 35.0 |  | 147.1 | 27.1 |
|  | 8 h post |  | 8.00 | |  |  |  | 50.3412 | 50 |  | 0.5 | 0.1 |  | 79.0 | 15.8 |  | 106.7 | 21.2 |
|  | 24 h post |  | 24.17 | |  |  |  | 45.7078 | 45 |  | traces^1^ | traces^1^ |  | 139.8 | 9.6 |  | 9.2 | 0.6 |
| U VII^c^ | pre |  | | -0.25 |  | 3.0392 |  | 50.3256 | 50 |  | traces^1^ | traces^1^ |  | 3.4 | 1.0 |  | 7.0 | 2.0 |
|  | 0.5 h post |  | | 0.50 |  |  |  | 50.065 | 50 |  | 0.6 | 0.5 |  | 133.2 | 125.0 |  | 44.1 | 41.3 |
|  | 1 h post |  | | 1.08 |  |  |  | 50.0377 | 50 |  | 1.3 | 0.8 |  | 239.4 | 148.3 |  | 125.9 | 78.0 |
|  | 2 h post |  | | 2.00 |  |  |  | 49.98 | 50 |  | 0.8 | 0.8 |  | 158.6 | 162.5 |  | 139.9 | 143.4 |
|  | 4 h post |  | | 4.00 |  |  |  | 50.0792 | 50 |  | 0.6 | 0.3 |  | 145.1 | 90.0 |  | 170.5 | 105.6 |
|  | 6 h post |  | | 6.00 |  |  |  | 50.2575 | 50 |  | 0.7 | 0.2 |  | 123.7 | 40.0 |  | 203.6 | 65.5 |
|  | 8 h post |  | | 8.00 |  |  |  | 50.0571 | 50 |  | 0.4 | 0.2 |  | 58.6 | 30.1 |  | 147.2 | 75.5 |
|  | 24 h post |  | | 24.00 |  |  |  | 50.0853 | 50 |  | traces^1^ | traces^1^ |  | 5.9 | 1.6 |  | 15.7 | 4.2 |

Tabelle S1: continued

| U VIII^c^ | pre |  | -0.33 |  | 3.0725 |  | 50.4058 | 50 |  | 0.5 | 0.1 |  | 61.4 | 13.8 |  | 97.6 | 21.82 |
| --- | --- | --- | --- | --- | --- | --- | --- | --- | --- | --- | --- | --- | --- | --- | --- | --- | --- |
|  | 0.5 h post |  | 0.52 |  |  |  | 49.9773 | 50 |  | 1.9 | 2.3 |  | 184.6 | 234.8 |  | 112.9 | 143.6 |
|  | 1 h post |  | 1.08 |  |  |  | 50.1023 | 50 |  | 1.9 | 1.1 |  | 423.9 | 255.5 |  | 256.0 | 154.0 |
|  | 2 h post |  | 2.08 |  |  |  | 50.0198 | 50 |  | 1.5 | 1.4 |  | 293.8 | 277.9 |  | 301.1 | 284.7 |
|  | 4 h post |  | 4.07 |  |  |  | 50.13 | 50 |  | 1.6 | 0.7 |  | 239.2 | 113.3 |  | 322.0 | 152.2 |
|  | 6 h post |  | 6.08 |  |  |  | 50.2297 | 50 |  | 1.3 | 0.3 |  | 197.8 | 52.6 |  | 348.3 | 92.2 |
|  | 8 h post |  | 7.90 |  |  |  | 50.1476 | 50 |  | 0.8 | 0.3 |  | 109.7 | 40.0 |  | 255.0 | 92.7 |
|  | 24 h post |  | 23.83 |  |  |  | 50.1662 | 50 |  | 0.3 | 0.1 |  | 26.2 | 9.9 |  | 48.3 | 18.1 |
| U IX^c^ | pre |  | -0.08 |  | 3.0618 |  | 45.1099 | 45 |  | < LOD^1^ | < LOD^1^ |  | < LOD^2^ | < LOD^2^ |  | < LOD^3^ | < LOD^3^ |
|  | 0.5 h post |  | 0.67 |  |  |  | 49.9356 | 50 |  | 1.0 | 0.9 |  | 164.4 | 156.1 |  | 54.8 | 52.1 |
|  | 1 h post |  | 1.17 |  |  |  | 49.9307 | 50 |  | 1.5 | 1.4 |  | 332.3 | 344.1 |  | 161.7 | 167.7 |
|  | 2 h post |  | 1.83 |  |  |  | 49.9366 | 50 |  | 1.4 | 1.2 |  | 337.6 | 320.5 |  | 248.6 | 236.2 |
|  | 4 h post |  | 4.17 |  |  |  | 50.1227 | 50 |  | 2.5 | 0.5 |  | 293.5 | 56.6 |  | 367.0 | 70.5 |
|  | 6 h post |  | 6.08 |  |  |  | 50.0436 | 50 |  | 1.7 | 0.5 |  | 182.9 | 57.5 |  | 343.5 | 107.8 |
|  | 8 h post |  | 8.08 |  |  |  | 50.6153 | 50 |  | 0.8 | 0.1 |  | 100.1 | 7.2 |  | 269.3 | 19.2 |
|  | 24 h post |  | 24.00 |  |  |  | 50.3217 | 50 |  | traces^1^ | traces^1^ |  | 30.4 | 3.5 |  | 5.9 | 0.7 |
| U X^d^ | pre |  | -0.07 |  | 3.0653 |  | 51.0938 | 50 |  | < LOD^1^ | < LOD^1^ |  | < LOD^2^ | < LOD^2^ |  | < LOD^3^ | < LOD^3^ |
|  | 0.5 h post |  | 0.53 |  |  |  | 50.0406 | 50 |  | 0.4 | 0.2 |  | 73.7 | 38.2 |  | 15.5 | 8.0 |
|  | 1 h post |  | 1.03 |  |  |  | 49.984 | 50 |  | 0.4 | 0.5 |  | 77.0 | 97.5 |  | 27.7 | 35.2 |
|  | 2 h post |  | 2.03 |  |  |  | 49.9557 | 50 |  | 0.3 | 0.5 |  | 68.8 | 102.4 |  | 48.6 | 72.3 |
|  | 4 h post |  | 3.85 |  |  |  | 50.0306 | 50 |  | 0.4 | 0.3 |  | 68.2 | 51.9 |  | 79.1 | 60.1 |
|  | 6 h post |  | 5.80 |  |  |  | 50.2313 | 50 |  | 0.5 | 0.2 |  | 68.6 | 29.1 |  | 89.3 | 37.7 |
|  | 8 h post |  | 7.75 |  |  |  | 50.1981 | 50 |  | 0.3 | 0.1 |  | 37.9 | 16.3 |  | 85.2 | 36.5 |
|  | 24 h post |  | 23.97 |  |  |  | 51.056 | 50 |  | 0.2 | 0.02 |  | 23.9 | 2.2 |  | 53.0 | 4.9 |

Tabelle S1: continued

| U XI^d^ | pre |  | -0.08 |  | 3.0818 |  | 50.1357 | | 50 |  | 0.8 | 0.2 |  | 3.8 | 1.1 |  | 9.0 | 2.6 |
| --- | --- | --- | --- | --- | --- | --- | --- | --- | --- | --- | --- | --- | --- | --- | --- | --- | --- | --- |
|  | 0.5 h post |  | 0.50 |  |  |  | 49.9494 | | 50 |  | 0.4 | 0.4 |  | 64.0 | 77.5 |  | 27.5 | 33.4 |
|  | 1 h post |  | 1.02 |  |  |  | 49.9495 | | 50 |  | 0.6 | 0.7 |  | 106.2 | 133.3 |  | 65.7 | 82.6 |
|  | 2 h post |  | 2.00 |  |  |  | 49.9904 | | 50 |  | 0.5 | 0.7 |  | 102.2 | 133.3 |  | 93.5 | 122.1 |
|  | 4 h post |  | 4.00 |  |  |  | 50.2611 | | 50 |  | 0.7 | 0.3 |  | 109.5 | 41.5 |  | 130.9 | 49.4 |
|  | 6 h post |  | 5.93 |  |  |  | 50.79 | | 50 |  | 0.5 | 0.1 |  | 60.3 | 9.3 |  | 118.8 | 18.0 |
|  | 8 h post |  | 8.00 |  |  |  | 50.2452 | | 50 |  | 0.3 | 0.1 |  | 29.8 | 13.0 |  | 88.0 | 38.3 |
|  | 24 h post |  | 24.00 |  |  |  | 50.7194 | | 50 |  | traces^1^ | traces^1^ |  | 7.2 | 1.0 |  | 18.6 | 2.6 |
| U XII^d^ | pre |  | -0.08 |  | 3.0324 |  | 40.644 | | 40 |  | traces^1^ | traces^1^ |  | 3.9 | 0.2 |  | 2.0 | 0.1 |
|  | 0.5 h post |  | 0.48 |  |  |  | 50.473 | | 50 |  | 0.3 | 0.04 |  | 83.8 | 11.2 |  | 17.3 | 2.3 |
|  | 1 h post |  | 1.03 |  |  |  | 49.9574 | | 50 |  | 0.3 | 0.3 |  | 82.1 | 83.2 |  | 33.1 | 33.6 |
|  | 2 h post |  | 1.85 |  |  |  | 49.9338 | | 50 |  | 0.9 | 1.3 |  | 72.2 | 108.2 |  | 48.2 | 72.4 |
|  | 4 h post |  | 3.80 |  |  |  | 50.2196 | | 50 |  | 0.5 | 0.2 |  | 84.6 | 31.9 |  | 76.1 | 28.5 |
|  | 6 h post |  | 5.82 |  |  |  | 50.6096 | | 50 |  | 0.4 | 0.1 |  | 61.6 | 10.8 |  | 82.8 | 14.3 |
|  | 8 h post |  | 7.82 |  |  |  | 50.194 | | 50 |  | 0.3 | 0.1 |  | 28.7 | 10.1 |  | 61.4 | 21.6 |
|  | 24 h post |  | 23.92 |  |  |  | 51.0036 | | 50 |  | traces^1^ | traces^1^ |  | 11.3 | 0.8 |  | 6.6 | 0.4 |
| U XIII^e^ | pre |  | -0.08 |  | 3.0533 | |  | 50.0408 | 50 |  | < LOD^1^ | < LOD^1^ |  | < LOD^2^ | < LOD^2^ |  | < LOD^3^ | < LOD^3^ |
|  | 0.5 h post |  | 0.42 |  |  | |  | 50.0013 | 50 |  | 0.2 | 0.1 |  | 38.0 | 23.8 |  | 19.8 | 12.4 |
|  | 1 h post |  | 0.92 |  |  | |  | 50.2214 | 50 |  | 0.9 | 0.3 |  | 216.6 | 87.7 |  | 145.0 | 58.5 |
|  | 2 h post |  | 1.92 |  |  | |  | 50.3107 | 50 |  | 1.0 | 0.3 |  | 197.5 | 57.1 |  | 182.5 | 52.4 |
|  | 4 h post |  | 3.92 |  |  | |  | 50.4126 | 50 |  | 0.8 | 0.2 |  | 105.6 | 22.5 |  | 174.3 | 36.8 |
|  | 6 h post |  | 5.92 |  |  | |  | 50.9174 | 50 |  | 0.4 | 0.04 |  | 42.9 | 3.7 |  | 105.0 | 9.0 |
|  | 8 h post |  | 7.92 |  |  | |  | 50.7388 | 50 |  | 0.3 | 0.03 |  | 2.6 | 0.3 |  | 67.5 | 7.7 |
|  | 24 h post |  | 23.92 |  |  | |  | 50.7838 | 50 |  | traces^1^ | traces^1^ |  | 2.6 | 0.3 |  | 8.5 | 1.0 |

Tabelle S1: continued

| U XIV^e^ | pre |  | -0.08 |  | 2.9119 |  | 50.946 | 50 |  | < LOD^1^ | < LOD^1^ |  | < LOD^2^ | < LOD^2^ |  | < LOD^3^ | < LOD^3^ |
| --- | --- | --- | --- | --- | --- | --- | --- | --- | --- | --- | --- | --- | --- | --- | --- | --- | --- |
|  | 0.5 h post |  | 0.67 |  |  |  | 25.4977 | 25 |  | 0.4 | 0.02 |  | 115.6 | 7.0 |  | 24.0 | 1.4 |
|  | 1 h post |  | 1.08 |  |  |  | 45.1345 | 45 |  | 1.0 | 0.2 |  | 166.6 | 43.0 |  | 62.5 | 16.1 |
|  | 2 h post |  | 1.75 |  |  |  | 49.9555 | 50 |  | 0.4 | 0.4 |  | 87.2 | 84.7 |  | 62.4 | 60.7 |
|  | 4 h post |  | 4.08 |  |  |  | 50.4278 | 50 |  | 0.6 | 0.1 |  | 84.2 | 13.9 |  | 91.0 | 14.9 |
|  | 6 h post |  | 6.00 |  |  |  | 50.0201 | 50 |  | 0.3 | 0.1 |  | 40.7 | 23.0 |  | 76.3 | 43.2 |
|  | 8 h post |  | 8.08 |  |  |  | 50.6635 | 50 |  | 0.3 | 0.02 |  | 28.2 | 2.5 |  | 66.2 | 5.9 |
|  | 24 h post |  | 23.83 |  |  |  | 50.7185 | 50 |  | traces^1^ | traces^1^ |  | 3.0 | 0.2 |  | 9.1 | 0.5 |
| U XV^e^ | pre |  | -0.12 |  | 3.0942 |  | 50.0656 | 50 |  | < LOD^1^ | < LOD^1^ |  | < LOD^2^ | < LOD^2^ |  | < LOD^3^ | < LOD^3^ |
|  | 0.5 h post |  | 0.38 |  |  |  | 49.8798 | 50 |  | 0.4 | 0.3 |  | 54.4 | 43.7 |  | 17.0 | 13.7 |
|  | 1 h post |  | 0.88 |  |  |  | 49.9389 | 50 |  | 1.2 | 1.0 |  | 177.5 | 150.4 |  | 82.5 | 70.0 |
|  | 2 h post |  | 1.88 |  |  |  | 49.9658 | 50 |  | 1.3 | 0.8 |  | 179.8 | 111.4 |  | 134.0 | 83.1 |
|  | 4 h post |  | 3.92 |  |  |  | 50.6829 | 50 |  | 2.3 | 0.2 |  | 154.5 | 13.2 |  | 198.1 | 16.7 |
|  | 6 h post |  | 5.88 |  |  |  | 50.0104 | 50 |  | 0.5 | 0.2 |  | 35.2 | 16.2 |  | 109.8 | 50.6 |
|  | 8 h post |  | 7.88 |  |  |  | 50.1256 | 50 |  | 0.3 | 0.1 |  | 16.9 | 4.8 |  | 71.4 | 20.4 |
|  | 24 h post |  | 23.92 |  |  |  | 50.1761 | 50 |  | traces^1^ | traces^1^ |  | 3.4 | 0.9 |  | 3.6 | 0.9 |
| U XVI^e^ | pre |  | -0.08 |  | 3.0253 |  | 50.8797 | 50 |  | traces^1^ | traces^1^ |  | 2.4 | 0.2 |  | traces^3^ | traces^3^ |
|  | 0.5 h post |  | 0.82 |  |  |  | 40.8054 | 40 |  | 0.3 | 0.03 |  | 106.1 | 10.0 |  | 20.5 | 1.9 |
|  | 1 h post |  | 1.27 |  |  |  | 25.4042 | 25 |  | 0.6 | 0.1 |  | 209.2 | 24.8 |  | 67.0 | 7.8 |
|  | 2 h post |  | 2.00 |  |  |  | 50.3646 | 50 |  | 1.4 | 0.3 |  | 244.3 | 50.1 |  | 117.8 | 24.0 |
|  | 4 h post |  | 4.00 |  |  |  | 50.2285 | 50 |  | 1.1 | 0.3 |  | 168.9 | 46.7 |  | 135.0 | 37.2 |
|  | 6 h post |  | 6.02 |  |  |  | 50.6708 | 50 |  | 1.1 | 0.1 |  | 132.1 | 15.6 |  | 163.2 | 19.0 |
|  | 8 h post |  | 8.00 |  |  |  | 45.9716 | 45 |  | 0.6 | 0.03 |  | 66.3 | 3.9 |  | 125.3 | 7.3 |
|  | 24 h post |  | 23.92 |  |  |  | 50.3564 | 50 |  | traces^1^ | traces^1^ |  | 13.1 | 1.5 |  | 17.9 | 2.1 |

Tabelle S1: continued

| U XVII^f^ | pre |  | -0.08 |  | 3.1559 |  | 50.9404 | 50 |  | traces^1^ | traces^1^ |  | 5.4 | 0.3 |  | traces^3^ | traces^3^ |
| --- | --- | --- | --- | --- | --- | --- | --- | --- | --- | --- | --- | --- | --- | --- | --- | --- | --- |
|  | 0.5 h post |  | 0.58 |  |  |  | 35.7235 | 35 |  | 0.3 | 0.01 |  | 16.9 | 0.8 |  | 5.4 | 0.3 |
|  | 1 h post |  | 1.00 |  |  |  | 20.2158 | 20 |  | 0.9 | 0.1 |  | 118.8 | 10.7 |  | 52.8 | 4.7 |
|  | 2 h post |  | 2.17 |  |  |  | 50.0404 | 50 |  | 0.8 | 0.3 |  | 65.2 | 27.6 |  | 75.9 | 32.1 |
|  | 4 h post |  | 4.00 |  |  |  | 50.0873 | 50 |  | 0.6 | 0.2 |  | 42.3 | 15.5 |  | 75.3 | 27.6 |
|  | 6 h post |  | 6.08 |  |  |  | 50.2347 | 50 |  | 0.4 | 0.02 |  | 28.6 | 1.2 |  | 70.3 | 3.0 |
|  | 8 h post |  | 8.00 |  |  |  | 19.4684 | 19 |  | 0.4 | 0.02 |  | 25.6 | 1.8 |  | 73.1 | 5.1 |
|  | 24 h post |  | 23.92 |  |  |  | 45.8907 | 45 |  | traces^1^ | traces^1^ |  | 4.4 | 0.2 |  | 15.0 | 0.7 |
| U XVIII^f^ | pre |  | -0.08 |  | 3.1981 |  | 40.9308 | 40 |  | < LOD^1^ | < LOD^1^ |  | < LOD^2^ | < LOD^2^ |  | < LOD^3^ | < LOD^3^ |
|  | 0.5 h post |  | 0.63 |  |  |  | 19.3623 | 19 |  | 0.6 | 0.2 |  | 147.4 | 44.1 |  | 49.2 | 14.7 |
|  | 1 h post |  | 1.05 |  |  |  | 50.2161 | 50 |  | 0.6 | 0.3 |  | 142.3 | 84.8 |  | 68.1 | 40.5 |
|  | 2 h post |  | 2.00 |  |  |  | 50.0415 | 50 |  | 0.8 | 0.6 |  | 131.3 | 94.9 |  | 94.3 | 68.1 |
|  | 4 h post |  | 3.97 |  |  |  | 50.0265 | 50 |  | 0.8 | 0.4 |  | 112.3 | 50.9 |  | 124.7 | 56.4 |
|  | 6 h post |  | 5.97 |  |  |  | 50.1204 | 50 |  | 0.7 | 0.1 |  | 73.7 | 16.6 |  | 117.8 | 26.3 |
|  | 8 h post |  | 8.30 |  |  |  | 50.2979 | 50 |  | 1.0 | 0.4 |  | 36.6 | 17.1 |  | 90.7 | 42.2 |
|  | 24 h post |  | 24.13 |  |  |  | 50.1197 | 50 |  | traces^1^ | traces^1^ |  | 5.3 | 0.5 |  | 13.0 | 1.3 |
| U XIX^f^ | pre |  | -0.07 |  | 3.1133 |  | 20.3401 | 20 |  | < LOD^1^ | < LOD^1^ |  | < LOD^2^ | < LOD^2^ |  | < LOD^3^ | < LOD^3^ |
|  | 0.5 h post |  | 0.50 |  |  |  | 19.4816 | 19 |  | 0.6 | 0.1 |  | 117.7 | 15.1 |  | 34.7 | 4.4 |
|  | 1 h post |  | 1.00 |  |  |  | 20.2355 | 20 |  | 2.3 | 1.5 |  | 102.0 | 68.8 |  | 61.1 | 41.2 |
|  | 2 h post |  | 2.00 |  |  |  | 50.0218 | 50 |  | 0.4 | 0.4 |  | 72.6 | 70.6 |  | 79.8 | 77.6 |
|  | 4 h post |  | 4.00 |  |  |  | 49.9698 | 50 |  | 0.4 | 0.2 |  | 55.3 | 27.0 |  | 85.0 | 41.4 |
|  | 6 h post |  | 6.20 |  |  |  | 50.0745 | 50 |  | 0.3 | 0.1 |  | 21.9 | 9.8 |  | 68.2 | 30.7 |
|  | 8 h post |  | 7.93 |  |  |  | 50.0546 | 50 |  | 0.2 | 0.1 |  | 10.7 | 5.9 |  | 43.7 | 24.3 |
|  | 24 h post |  | 23.98 |  |  |  | 50.022 | 50 |  | 0.2 | 0.02 |  | 1.5 | 0.2 |  | 17.8 | 1.8 |
| ^1^ LOD (AMS): 1.6 ng, LOQ (AMS): 7.6 ng ^2^ LOD (AMSO): 22.0 ng, LOQ (AMSO): 77.4 ng ^3^ LOD (AMSO_2_): 22.4 ng, LOQ (AMSO_2_): 78.6 ng ^a^ … ^f^: same letters correspond to test persons that consumed garlic samples from the same garlic bulb | | | | | | | | | | | | |  |  |  |  |  |
